# Immune reconstitution following umbilical cord blood transplantation: IRES, a study of UK paediatric patients

**DOI:** 10.1002/jha2.12

**Published:** 2020-05-21

**Authors:** John Girdlestone, Meera Raymond, Bronwen Shaw, Sameer Tulpule, Vikesh R. Devlia, Robert Danby, Trudy Ahyee, Aurore Saudemont, Rachael Hough, Paul Veys, Annalisa Ruggeri, Ajay Vora, David I. Marks, Brenda Gibson, Robert Wynn, Alejandro Madrigal, Cristina V. Navarrete

**Affiliations:** ^1^ H&I R&D Group NHSBT Colindale Centre London UK; ^2^ Center for International Blood and Marrow Transplant Research Medical College of Wisconsin Milwaukee Wisconsin; ^3^ Department of Haematology Kokilaben Dhirubhai Ambani Hospital Mumbai India; ^4^ Department of Immunotherapy Anthony Nolan Research Institute London UK; ^5^ Department of Haematology University College London Hospitals London UK; ^6^ Bone Marrow Transplant Unit Great Ormond Street Hospital London UK; ^7^ Eurocord Paris France; ^8^ Bristol Haematology and Oncology Centre University Hospitals Bristol Bristol UK; ^9^ Paediatric Haematology Royal Hospital for Sick Children Glasgow UK; ^10^ Paediatric Bone Marrow Transplant Programme Royal Manchester Children's Hospital Manchester UK

**Keywords:** cord blood, immunophenotyping, stem cell transplantation

## Abstract

To obtain a qualitative as well as quantitative view immune reconstitution following umbilical cord blood (UCB) transplantation of paediatric patients, we utilised a broad panel of flow cytometry markers to monitor the phenotypes of lymphoid and myeloid cells at 1‐12 months post‐transplant. Samples were received from 46 patients with a median age of 3.3 years and survival was 76% at 1 year. Monocytes were at similar or higher median levels than in adult controls at all times tested, with a high CD16+ proportion in the first 3 months. NK cells were also within adult ranges, with a CD56++ high proportion in the first 6 months. B cell recovery was seen from 2 months in most patients and T cells from 3 months, both were delayed with anti‐thymocyte globulin (ATG) treatment. CD4:CD8 ratios were high in the first 6 months, and the proportion of T cells with recent thymic emigrant and naïve phenotypes rose from 3 months. NK and plasmacytoid dendritic cell numbers remained at reduced levels in patients not surviving to 1 year. Our results can serve as a useful reference for detailed monitoring of immune reconstitution in paediatric recipients of UCB.

## 1 INTRODUCTION

Umbilical cord blood (UCB) has proven to be an important alternative to adult sources of haematopoietic stem cells (HSC) for allogeneic transplantation, particularly for paediatric patients and for those with no suitable HLA‐matched donor [1]. The use of UCB can provide outcomes equivalent to those for adult bone marrow (BM) and peripheral blood HSC for a number of indications, but the main drawbacks have been limiting cell content and higher rates of graft failure and non‐relapse mortality associated with slower engraftment [1, 2, 3, 4]. Accumulating evidence has indicated the important interplay between HLA matching and TNC dose for successful outcomes, with optimal engraftment and survival rates achieved with TNC doses >2.5 × 10^7^/kg and allele level matching [5, 6]. Although the degree of HLA matching has generally been lower with UCB grafts, higher stringency is reported to reduce rates of acute Graft‐versus‐Host Disease (aGvHD) [5] and non‐relapse mortality (NRM) [6], while increasing relapse rates [7].

Increased rates of NRM with UCB transplants have been ascribed to slower engraftment, and also to the naïve state of the cells in the graft [4, 8]. Delays in the appearance of neutrophils, platelets, and other cells of the innate immune system following transplantation are associated with worse outcomes and even well‐matched cord grafts are relatively slow to engraft [5], although NRM does not increase significantly until the delay is beyond 42 days [2]. UCB recipients have higher rates of post‐transplant infections, particularly those with low levels of immune reconstitution, but this does not appear to be associated with increased NRM or survival [8, 9].

Neutrophil and platelet recovery remain the primary indicators of engraftment, but there is increasing evidence that the appearance of lymphoid and myeloid populations and sub‐populations can correlate with successful transplant outcomes [10, 11, 12, 13, 14, 15, 16, 17, 18, 19]. Although early indicators of engraftment tend to be delayed with cord blood, some lymphoid populations may appear more quickly and/or to higher levels than with adult sources and therefore compensate to some extent for other deficits [4, 10, 20]. Given the limiting numbers of T cells in a UCB graft, and their reduced propensity to cause GvHD, it has also been recognised that optimising the use of ATG during conditioning can provide for improved immune reconstitution [4, 11, 21].

In order to obtain a more comprehensive view of the dynamics of lymphoid and myeloid cell regeneration after UCB transplantation, we set up the Immune Reconstitution Study (IRES) in association with the Reduced Intensity Conditioning (RIC) and Myeloablative Conditioning (MAC) trials established to promote increased UCB usage in the United Kingdom [22]. Our immune panel was designed to capture as many relevant cell phenotypes as possible using a standard diagnostic lab flow cytometer, and our choice of markers coincides well with those of other immune monitoring studies [23, 24].

The study was designed to obtain information on the developmental and activation states of important lymphoid and myeloid populations with the aim of determining reference values for correlation with transplant outcomes.

## 2 METHODS

### 2.1 Patients and samples

Eligible patients were those under the age of 18 receiving an umbilical cord transplant in the United Kingdom from 2011 to 2017. One patient was included in the MAC trial (EUDRACT 2009‐011818‐21) administered by RH (UCLH). The protocol received national approval through a Research Ethics Committee (Riverside, 09/H0706/35) and individual transplant centres joined the study upon receiving approval by their local R&D committees. The patients were anonymous to the laboratory. Samples were obtained from a total of 46 patients, out of 49 recruited: Institute for Child Life & Health, University Hospitals Bristol; Sheffield Children's Hospital; Great Ormond Street Hospital, London; Royal Manchester Children's Hospital; Royal Hospital for Sick Children, Glasgow; University College London Hospitals; and The Royal Marsden Hospital, London. One patient was included in the MAC trial. Further samples were not collected from patients who relapsed. Evaluable samples received (eligible [alive, non‐relapsed] patients in parentheses): 31 at 1 month (46), 26 at 2 months (41), 27 at 3 months (39), 22 at 6 months (31), 15 at 12 months (27), and eight at 18‐24 months.

### 2.2 Flow cytometry

One‐millilitre samples of EDTA blood were routine for the younger patients, whereas larger volumes were obtained from the adolescents. Samples were transported to NHSBT and processed for whole blood (WB) staining within 48 h of drawing. Plasma samples were frozen if there was sufficient material. Incubations were performed with 30 µL WB made up to a final volume of 50 µL with antibody master mixes and PBS/2.5% Foetal Calf Serum (Gibco, UK). All antibodies were from BioLegend (London, UK) except for anti‐CCR7 (R&D Systems, Abingdon, UK). Antibodies and fluorophores are listed in Table 2. Absolute count beads (CountBright, ThermoFisher, UK) were added to tubes 1, 2, and 8, and 7‐aminoactinomycin D (7AAD) was used to determine cell viability in tube 13. Anonymous control samples were run in parallel, either cord blood (UCB) from redundant units provided by the NHS Cord Blood Bank, Colindale, or adult blood (AB) from healthy donors provided by the Testing Department, NHSBT Colindale. Samples were incubated with antibodies at room temperature for 20 min, and then lysed for 15' with 250 µL 1.2% ammonium oxalate (Sigma, Gillingham, UK) before running on a FacsCanto flow cytometer (Becton Dickinson [BD], Reading, UK). Analysis of cell populations was performed with Infinicyt (Cytognos, Salamanca, Spain) and FacsDiva (BD) software. Granulocytes were defined according to forward/side scatter, and antibody‐positive populations were determined in relation to the blank tube of unstained blood, and on negative populations within a stained sample (eg, CD19 on non‐B lymphocytes).

Clinical information for patients was provided by Eurocord. Statistics and plotting were performed in Prism (GraphPad, La Jolla, CA, USA) and Excel (Microsoft, Seattle, USA). Student's unpaired *t*‐test with Welch's correction was used for statistical comparison of cell populations, the Mann‐Witney non‐parametric test was used for GvHD comparisons, and a Kaplan‐Meier plot was used for displaying survival. Confidence intervals and difference of means were calculated in Prism.

## 3 RESULTS

**FIGURE 1:**
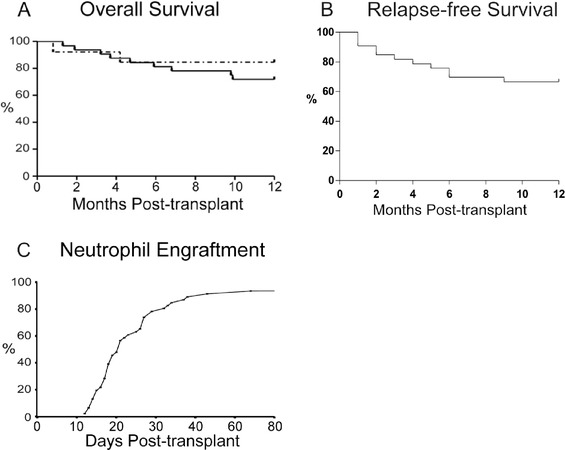
A, Overall survival of paediatric cohort. MD solid line (73%: 24 alive at one year out of 33 patients), non‐MD dotted line (85%: 11/13). B, Relapse‐free survival of patients treated for MD. C, Neutrophil engraftment (93%: 43/46), median 20.5 days

The clinical and demographic characteristics of the study cohort are presented in Table 1. The 1‐year survival was 76%; in the patients with malignant disease (MD), there were five deaths due to relapse and four to NRM, both non‐malignant deaths were NRM. There was no neutrophil engraftment by 100 days for three patients, although all were alive at 1 year (Figure 1B). A higher proportion of males were CMV+ at transplant, and this did not appear to be due to a higher average age compared to the females and was not associated with higher mortality (M: 19/23 and F: 16/23 alive at 1 year). Recipients above the median age (3.3 years) were more likely to have MD (12/23 vs 21/23; *P* = .003) and to have received TBI (1/22 vs 9/21; *P* = .002).

**TABLE 1:** Characteristics of paediatric cord blood recipients in study

Patients	46*
Age	Median 3.3, range 0.3‐17.1
1 year survival	76% (73% MD, 85% non‐MD)
Gender	Female 50%
Disease	
Malignant	29 Acute leukaemia 1 myelodysplastic syndrome (MDS) 3 combined MDS/myeloproliferative disorder
Non‐malignant	1 histiocytosis 2 bone marrow failure 4 inborn error of metabolism 1 autoimmune disease 5 primary immune deficiency
Conditioning	MAC 59%
Cords	Singles 91% (4 doubles, >12 years of age)
TNC	Median dose 8.1 × 10^7^/kg (range 2.3‐32)**
Relapse	15% within 12 M of those treated for malignancy
NRM	11% within 12 M
Engraftment	Neutrophil 93% by 100 days, median 20.5 days
CMV^pos^ at tx	62% male, 18% female (*P* < .005)
SeroATG	14 of 46 (31%)
HLA Match	Serological A, B, C, D: 8 × 8/8, 11 × 7/8, 9 × 6/8, 5 × 5/8, 2 × 4/8
aGvHD	Grade 0, 10.5%; 1, 21%; 2, 55%; 3, 5%; 4, 8%
cGvHD	20%

* A further three patients were enrolled but no samples were received.

** Same median value was seen for ‘TNC collected’ and ‘TNC infused’.

### 3.1 Enumeration and characterisation of white blood cell populations

The main aim of this study was to monitor the dynamics of immune cell populations following UCB transplantation, and the antibody panel for capturing their surface marker expression is shown in Table 2. Tube 1 was used to quantify the main lymphocyte populations, as well as monocytes and their CD16+ subset. Tube 2 was included to follow circulating haematopoietic progenitor and stem cells but numbers were very low, below those found in average adult samples, and no further analysis was performed. T cells with a regulatory (CD3+CD4+CD25+CD127low) and Recent Thymic Emigrant (RTE) phenotypes (CD3+CD45RA+CD31+) were identified with Tube 3 [25]. Further markers of T cell maturation and function were used in Tubes 6, 7, 10, and 12. The TCR usage of T and NKT cells was measured with Tube 4, but there was a very low rate of gamma‐delta positive cells and no further analysis was performed. Antibodies against CD8 were not included for all T cell analyses but results from tube 6 showed that there were negligible numbers of double negative CD3+ cells (data not shown), so CD3+CD4‐ cells were considered to be CD8+. A few samples contained T cells with significant proportions that were positive for Cutaneous Lymphoid Antigen (CLA). Correspondence with the transplant centres indicated that the patients had GvHD or infections, but more detailed correlation with clinical events would be required to determine if CLA or homing receptors could be used as informative markers. B cell markers were included in Tubes 9 and 11, and we concentrated on CD24hiCD38hi Transitional B (TrB), and CD27+ memory B for this analysis. Myeloid and plasmacytoid dendritic cells were analysed with tube 8. In addition to Tube 1, NK subsets were detected with Tube 12.

**TABLE 2:** Staining panel for flow cytometry

Tube	Type	Fitc	PE	PerCp	PE‐Cy7	APC	APC‐Cy7
1	Mono, B, T NK, NK‐T	14	56	3	45	19	16
2	Stem	DR	117	34	38	133	45
3	Treg/RTE	45RA	31	3	25	127	4
4	TCR	γδ	αβ	3	25	56	4
5	Homing	CLA	CXCR4	CCR6	3	CXCR3	4
6	T stages	45RA	CCR7	3	25	8	4
7	Activation	HLA‐DR	69	3	25	14	4
8	DC	1c	11c	DR	123	304	19
9	B stage 1	24	21	38	10	27	19
10	Effector T	28	56	161	3	27	4
11	B stage 2	43	27	DR	20	5	19
12	NK	57	94	3	62L	56	16
13	Viability	‐	‐	7AAD	‐	‐	45
14	Blank	‐	‐	‐	‐	‐	‐

*Note*. Numbers refer to CD nomenclature. The blank tube contained an unstained blood sample and was used to set baselines, and adjust FSC/SSC if necessary. 7AAD exclusion was used to determine if there were acceptable levels of viability in the gated CD45+ populations (>90% for lymphoid cells). Mono: Monocyte, B; B lymphocyte, T: T lymphocyteTreg: T Regulatory Cell, TCR: T Cell Receptor, DC: Dendritic Cell, NK: Ntural Killer lymphocyte.

**FIGURE 2:**
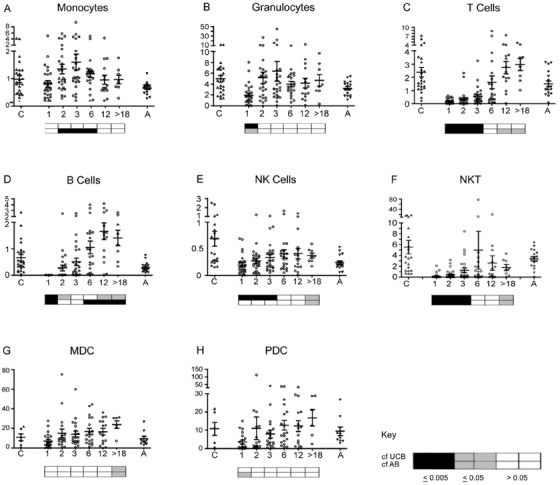
Absolute cell counts for the main white blood cell populations. Flow cytometry was performed on whole blood with counting beads to determine the number of cells /mL for (A) monocytes (CD14+), (B) granulocytes (FSC/SSC), (C) T cells (CD3+), (D) B cells (CD19+), (E) NK cells (CD56++ and CD56+/16+), (F) NKT cells (CD3+ CD56+), (G) Myeloid dendritic cells (HLA‐DR+ CD19‐ CD1c+ CD11c+), and (H) plasmacytoid dendritic cells (PDC: HLA‐DR+ CD123+ CD304+). The *Y*‐axes are 10^6^ cells/mL for A‐E, 10^4^ cells/mL for F, and 10^3^ cells/mL for G‐H and the *X*‐axes are months after transplant. The ‘>18’ group includes eight patients sampled at 18‐36 months. Values for umbilical cord blood (C) and adult peripheral blood (A) controls are provided for reference. The FACS files for outlying samples were reanalysed for confirmation. Horizontal bars represent means with standard error. Key: The boxes below the plots represent *t*‐test comparisons against the values for UCB (upper row) and AB (bottom row): white *P* > 0.5; grey *P* ≤ .05; black *P* ≤ .005. UCB and AB differed significantly (*P* < .05) for cell types in (A‐E). Further statistical information in Table S1

Most patients had granulocytes within normal ranges by 1‐2 months (Figure 2) and recipients below the median age had relatively higher levels at 6 and 12 months (Figure S1). Monocytes were above the healthy adult mean from 2 to 6 months and then returned to lower levels. An apparent overshoot of B and T cell numbers compared to average AB concentrations was observed; however, the higher levels are consistent with reference median values for healthy children [26] (see also Figure S1). There was no significant difference between the recovery of CD4 and CD8 T cells, although the CD4:CD8 ratio declined over the first year (Figure S2). For most patients, NK cells remained within normal adult ranges during the first year, whereas NKT were generally low but with some outliers at 3‐12 months. Dendritic cell numbers tended to increase with time but in the first year remained within ranges seen for adult controls, with PDC showing greater variation.

**FIGURE 3:**
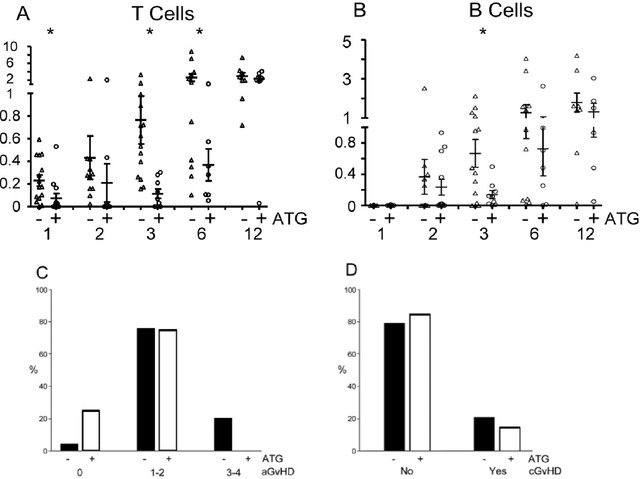
ATG use. (A) T cell and (B) B cell numbers (10^6^ /mL) at 1‐12 months post‐transplant according to ATG use. Percentage of patients grades of aGvHD (C) and development of cGvHD (D) by ATG use. Asterisks indicate *P* < .05. Further statistical information for A and B in Table S2

The use of ATG in the conditioning regimen was associated with lower T cell numbers at 1‐6 months in comparison with untreated patients (Figure 3A). T cell numbers had started to rise by 3 months without ATG (cf. 1 month), whereas significant increases were not seen until 12 months in the treated patients. B cell numbers were also significantly lower at 3 months (Figure 3B). The proportion of T cells with an RTE or naïve phenotype at 3‐12 months did not differ according to ATG use (data not shown). The CD4:CD8 ratio was lower at 1 and 2 months in the few ATG‐treated patients where T cells were present (Figure S2C). ATG use was associated (*P* = .0125) with a decrease in aGvHD (III‐IV), but did not correlate with development of cGvHD (Figures 3C and 3D).

### 3.2 Lymphocyte maturation markers

**FIGURE 4:**
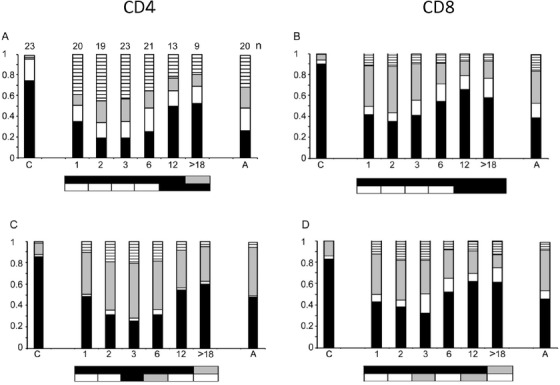
T Cell stages. RTE analysis of (A) CD3+ CD4+, (B) CD3+ CD4‐ cells: Relative proportions of CD45RA+ CD31+ (RTE, black); +/– (white), –/+ (grey), –/– (stripes). Maturation state analysis of (C) CD3+ CD4, (D) CD3+ CD8 cells: CD45RA+ CCR7+ (naïve, black), +/– (effector memory RA+, white), –/+ (central memory, grey), –/– (effector memory, stripes). ‘C’ cord blood and ‘A’ adult blood, horizontal axis numbers indicate months after transplant. The boxes below the plots represent *t*‐test comparisons against UCB and AB (as in Figure 2) for black bar sub‐populations: RTE (CD45RA+ CD31+) and naïve cells (CD45RA+ CCR7+). The number (n) of samples analysed for each set of markers is indicated above the bars in Figure 4A

In patients with detectable levels of T cells at 1 month post‐transplant, there were relatively low proportions of CD4 and CD8 T cells exhibiting RTE and naïve phenotypes, and the patterns of maturation and activation markers were similar to those seen in adults rather than cords (Figure 4). RTE proportions continued to decrease until 3 months, then increased until they were above those seen in adults by 12 months (Figures 4A and 4B). Naïve cell (CD45RA+ CCR7+) proportions at 3 months dropped below those seen in adults but then recovered by 12 months to similar (CD4) or greater (CD8) levels (Figures 4C and 4D). Therefore, the increase in T cell number from 3 to 6 months appears primarily due to the generation of new, thymically derived lymphocytes.

**FIGURE 5:**
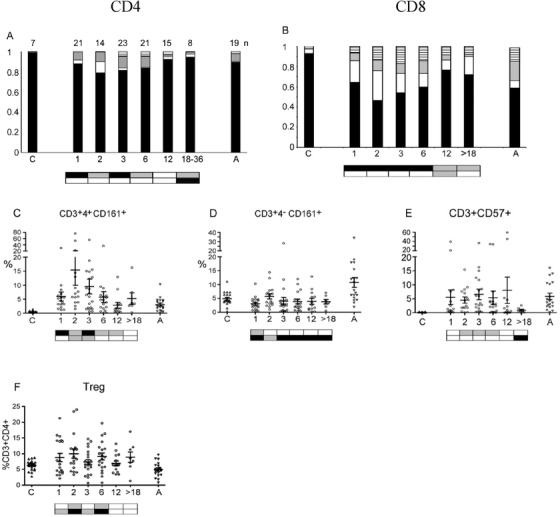
Functional T cell markers. Proportion of (A) CD4 and (B) CD8 cells expressing co‐stimulatory factors CD27 and CD28: CD27+CD28+ (black), +/– (white), –/+ (grey), –/– (stripes). The number (n) of samples analysed for each set of markers is indicated above the bars in Figure 5A. Percentage of (C) CD3+CD4+ and (D) CD3+CD4‐cells expressing CD161. (E) Total CD3 population expressing CD57. (F) T regulatory cells (CD3+4+25+127low). ‘C’ cord blood and ‘A’ adult blood; horizontal axis numbers indicate months after transplant. The boxes below the plots represent *t*‐tests against UCB (upper) and AB (lower), for A and B, the comparisons are for CD27+CD28+ (black bars). Further statistical information for (C‐F) in Table S3

Functional markers on T cells show substantial changes from UCB levels by 1 month post‐transplant (Figure 5). There were significant decreases in CD3+ cells expressing the co‐stimulatory membrane proteins CD27 and /or CD28, with the effect more pronounced in CD8+ cells (Figure 5B). The trend to a more naïve CD27+CD28+ pattern became evident from 3 to 6 months. The proportion of CD4+ CD161+ cells increased in the first few months and then decreased to AB levels by 6 months (Figures 5C and 5D). CD57 expression, characteristic of late stage effector memory T cells [27], was at AB levels in most patients by 1 month but dropped significantly after 1 year (Figure 5E). CD3+CD4+CD25+CD127lo T cells showed a great deal of variation among patients, and were relatively higher as a proportion of CD4+ cells at 2‐6 months (Figure 5F). Although this surface phenotype is characteristic of T regulatory cells, we cannot rule out the inclusion of activated effector cells given the difficulty of discriminating between them.

**FIGURE 6:**
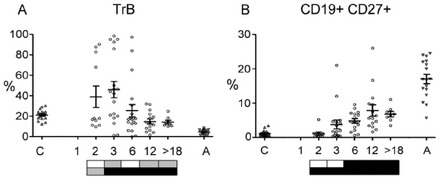
B Lymphocytes. The percentages of CD19+ B cells that have a (A) TrB (CD24hiCD38hi) or (B) memory (CD27+) phenotype. Further statistical information in Table S4

B lymphocytes are notable by their absence at 1 month after transplant, but in most patients they rebounded to adult levels by month 2 (see Figure 2D). The high proportion of TrB and low levels of memory B cells indicate that they are being newly generated in the marrow (Figure 6). The maturation profile appears to stabilise by 12 months with TrB higher, and memory B cells at lower levels than seen in adults.

### 3.3 Innate immune cells

**FIGURE 7:**
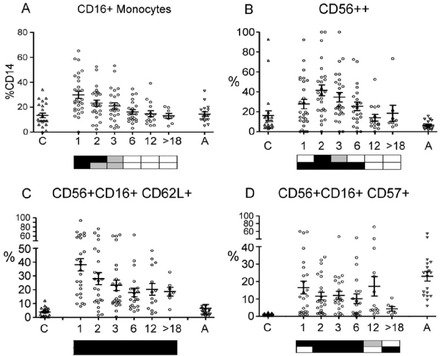
Innate immune cells. A, CD16+ monocytes as % of total CD14+ monocytes. B, CD56++ proportion of total NK cells. C, CD62L+ NK cells as a proportion of CD56+CD16+ cells. D, CD57+ NK cells as a proportion of CD56+CD16+ cells. Further statistical information in Table S5

Most patients had circulating monocytes similar to or higher than the adult mean within 1 month of transplant. The CD16+ subset was significantly higher than found in UCB or AB and then decreased by 6 months (Figure 7A). NK cells drop from the concentrations found in UCB but most patients were within adult ranges from 1 month post‐transplant (Figure 1), with age differences (Figure S1). Early NK cells (CD56++ CD16lo) are a significant proportion of the total NK population in the first 6 months after transplant (Figure 7B). Most patients had a high proportion of CD62L+ NK cells (Figure 7C), but a low CD57+ ratio (Figure 7D).

### 3.4 Age‐related differences

**FIGURE 8:**
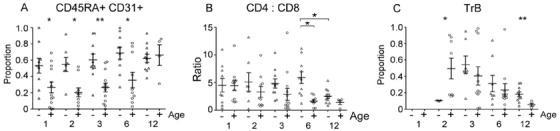
Age‐related differences in lymphocyte sub‐populations. A, CD3+ CD4– CD45RA+ 31+ cells as a proportion of CD3+ 4– by median age (– below, + above median). B, CD4:CD8 ratio. (C) TrB by median age as a proportion of total CD19+ lymphocytes. (*P < .05; **P < .005). Further statistical information in Table S6

Patients below the median age had higher levels of CD3 cells at 12 months (Figure S1A), whereas those above had lower levels of CD8 RTE until 12 months post‐transplant (Figure 8A). Granulocytes and NK cells were also higher at 12 months in the younger patients (Figures S1B and S1C). The CD4:CD8 ratio in the entire cohort was higher than the usual 2:1 for the first 6 months (Figure S2B), with younger patients showing a prolonged elevation (Figure 8B). Patients below the median age had a delayed appearance of TrB (Figure 8C), but higher levels of total B cells by 12 months (Figure S1D).

## 4 DISCUSSION

The 1 year survival rate of 76% for our cohort was favourable in relation to the outcomes for paediatric UCB transplants in the United Kingdom from 1998 to 2012 [28], and the Eurocord cohort from 1994 to 2011 [2]. Despite the relatively high TNC/kg that can be achieved with paediatric patients, the median time of 20.5 days for neutrophil engraftment was relatively slow compared to other SC sources [29]. Monocytes and NK were within or above normal range from 1 month, with granulocytes recovering by the second month after transplant. An elevated proportion of CD16+ monocytes was notable in the first few months after transplant, but the significance of intermediate and non‐classical monocytes [30] in immune reconstitution is unclear. The proportion of NK cells with a CD62L+ phenotype was also raised post‐transplant but further studies will be required to determine if this affects tissue‐homing patterns, and is due to the nature of the graft and/or responses to the paediatric post‐transplant milieu.

T cell reconstitution was delayed with the use of ATG, as noted by others [4]. ATG treatment was associated with a lower CD4:CD8 ratio and a delayed recovery in B cell numbers. Even with ATG, T and B numbers were above normal adult values by 12 months, consistent with reference counts for children [26]. The proportion of CD27+ (Memory) B cells remained significantly lower than seen in healthy adults which may be a corollary of the higher B cell numbers in children.

The proportions of RTE and naïve T cells dropped until 3 months indicating activation by alloantigens or pathogens, although lymphopaenia‐induced proliferation may also contribute [31]. These proportions started to increase from 3 months, indicating the net contribution of new cells from the thymus. Even within our cohort, there were age‐related differences, with those above the median showing a lower recovery of CD8 RTE. Further investigation will be needed to see if this correlates with nature of the patients’ disease, post‐transplant infections, or age‐related differences in homeostatic settings. The proportion of CD27+CD28+ T cells dropped over the first few months post‐transplant, particularly in the CD8 population (CD4‐ in our analysis) with a concomitant increase of CD27+CD28‐. CD27+28‐ ‘intermediate’ CD8 cells can represent the majority of virus‐specific cells in some cases [32]. By 1 year, the CD27+28+ cells had recovered and surpassed the proportion seen in healthy adults.

An inversion of the usual CD4:CD8 ratio is seen with BM transplantation and has also reported for CB transplants [33, 34], but in agreement with others we found the usual CD4 majority [3, 35]. Although a few patients with very high ratios skew the average, most had a value >1 at all time points. The ratio was significantly higher at early time points in the absence of ATG use. Until several years of age, healthy children have a 4:8 ratio >2 [26], and again it will be necessary to distinguish between age effects of the recipients and the intrinsic survival and recovery rates of the T lymphocyte subsets.

There was a notable increase in the proportion CD4+ T cells expressing of CD161+ post‐transplant. Th17 cells are known to express the C‐type lectin CD161, but it is also found on other T cell sub‐populations and further investigation would be required to characterise the cells [36, 37]. At each time point, some patients expressed relatively high levels of CD4 cells with a Treg surface phenotype, but without FoxP3 or other discriminating markers it is difficult to discount them being activated effector T cells.

Dendritic cells are crucial to initiating immune responses and influencing their nature. Low PDC levels post‐transplant have been associated previously with poor outcomes [13, 38], although high PDC have been correlated with increased relapse rates in paediatric recipients [12]. In our cohort, there was a wide variation in PDC levels between individuals and, although patient numbers were low, those with very low levels had poorer survival, with most succumbing to NRM rather than relapse (Figure S3). PDC are characterised by their stores of type I interferons, but it is not known if viral infections were the primary cause of mortality. NK cells, but not MDC or monocytes (Figure S3), were also reduced in the non‐survivors but it's conceivable that low PDC and NK numbers were associated with the use of steroids or other agents, rather than directly contributing to mortality.

The nature of immune reconstitution in a patient will be determined by the complex interplay among the stem, progenitor, and effector cells in the graft and the environment of the patient that has been subjected to conditioning, and post‐transplant treatments, infections, and potential immune complications such as GvHD. The power of our study was limited by the diversity of the patients and transplant protocols, but there were consistent patterns of reconstitution that may serve as references for UCB recipients and indicate avenues for future examination. The immune system undergoes substantial changes from the neonatal period through to puberty, and even within our paediatric cohort there were distinct age‐related patterns of cell numbers and phenotypes. The maturation profiles of T, B, and NK lymphocytes in the recipients at 1 year post‐transplant were distinct from those seen in healthy adults, but the relative contributions of the SC source and age‐related homeostatic settings need to be investigated. The relatively high CD4:CD8 ratio we observed is in contrast to the pattern seen with adult SC sources, and is likely to have fundamental consequences for immune behaviour following UCB transplantation. Although the absolute numbers of T cells are low in the first months after transplant, the proportions of some subsets, such as CD4+CD161+, are elevated and merit further investigation as to their significance. The analysis of immune cell subsets will be facilitated by the rapid development of high information content cytometric methods, which may allow for diagnostic and prognostic monitoring of SC recipients.

## AUTHOR CONTRIBUTIONS

JG, BES, ST, AM, RH, and CVN proposed and designed the study. JG, RD, and AS supervised the sample analysis. JG, MR, ST, and VRD performed sample analysis. TA organised regulatory affairs and hospital liaison. RH, PV, AV, DIM, BG, and RW provided patient material. AR provided patient data. JG wrote the manuscript. BES, RD, PV, AV, DIM, RW, AM, and CVN reviewed the manuscript.

## Supporting information

SUPPORTING INFORMATION

SUPPORTING INFORMATION

SUPPORTING INFORMATION

SUPPORTING INFORMATION

SUPPORTING INFORMATION

SUPPORTING INFORMATION

SUPPORTING INFORMATION

SUPPORTING INFORMATION

SUPPORTING INFORMATION

SUPPORTING INFORMATION
