# Supplementary material for: Immune reconstitution following umbilical cord blood transplantation: IRES, a study of UK paediatric patients
Source: EJHaem. 2020 May 21;1(1):208–18. doi: 10.1002/jha2.12 (PMC9176140; doi:10.1002/jha2.12)
Supplement: Supplementary file 1 — SUPPORTING INFORMATION [file JHA2-1-208-s007.pdf]

Sample	Cord		1	2	3	6	12	18-24	Month	Adult
**Monocytes Fig 2A**										
Number of values	24		30	26	26	22	15	8		17
Mean	816663		647476	1.17E+06	1.40E+06	997265	786321	796895		504036
Std. Deviation	566492		598764	856377	1.43E+06	606499	614811	430085		207269
Std. Error	115635		109319	167949	280802	129306	158743	152058		50270
Lower 95% CI of mean	577457		423890	822488	822680	728358	445847	437331		397468
Upper 95% CI of mean	1.06E+06		871061	1.51E+06	1.98E+06	1.27E+06	1.13E+06	1.16E+06		610604
Diff in mean cf Adult			143400	664400	897000	493200	282300	292900		
SE of diff			120300	175300	285300	138700	166500	160200		
95% CI diff		from	-99990	305800	310500	208500	-70720	-76450		
		to	386900	1023000	1483000	777900	635300	662200		
P			0.24	0.0007	0.0041	0.0014	0.11	0.10		
**Granulocytes Fig 2B**										
Number of values	24		30	26	26	22	15	8		17
Mean	4.98E+06		1.99E+06	5.16E+06	6.38E+06	4.01E+06	4.18E+06	4.69E+06		3.21E+06
Std. Deviation	2.95E+06		1.84E+06	5.29E+06	9.19E+06	2.19E+06	3.43E+06	3.09E+06		1.31E+06
Std. Error	601891		336523	1.04E+06	1.80E+06	467064	885538	1.09E+06		317052
Lower 95% CI of mean	3.74E+06		1.30E+06	3.03E+06	2.67E+06	3.03E+06	2.28E+06	2.11E+06		2.53E+06
Upper 95% CI of mean	6.23E+06		2.68E+06	7.30E+06	1.01E+07	4.98E+06	6.08E+06	7.27E+06		3.88E+06
Diff in mean cf Adult			-1215000	1956000	3178000	798100	975000	1482000		
SE of diff			462400	1084000	1830000	564500	940600	1136000		
95% CI diff		from	-2148000	-260600	-585000	-348700	-1010000	-1137000		
		to	-281200	4173000	6940000	1945000	2960000	4102000		
P			0.012	0.082	0.094	0.17	0.31	0.23		
**T Cells Fig 2C**										
Number of values	24		30	26	26	22	15	8		17
Mean	2.37E+06		154541	326420	554774	1.62E+06	2.73E+06	2.97E+06		1.49E+06
Std. Deviation	1.75E+06		181458	571744	700205	2.31E+06	1.80E+06	1.29E+06		939480
Std. Error	356219		33130	112128	137322	492462	464989	456311		227857
Lower 95% CI of mean	1.64E+06		86782	95488	271955	594647	1.73E+06	1.89E+06		1.01E+06
Upper 95% CI of mean	3.11E+06		222299	557353	837593	2.64E+06	3.72E+06	4.05E+06		1.98E+06
Diff in mean cf Adult			-1338000	-1166000	-937600	126400	1234000	1473000		
SE of diff			230300	254000	266000	542600	517800	510000		
95% CI diff		from	-1826000	-1691000	-1484000	-983300	153700	336800		
		to	-849700	-640500	-391700	1236000	2314000	2610000		
P			<0.0001	0.0001	0.0015	0.8175	0.0272	0.0162		
**B Cells Fig 2D**										
Number of values	24		30	26	26	22	15	8		17
Mean	659637		906	272334	504611	1.05E+06	1.66E+06	1.40E+06		289618
Std. Deviation	628890		2707	535232	699892	1.12E+06	1.22E+06	827922		187161
Std. Error	128372		494.2	104968	137260	239580	315804	292714		45393
Lower 95% CI of mean	394082		-104.7	56149	221919	550616	984260	712162		193388
Upper 95% CI of mean	925191		1917	488519	787304	1.55E+06	2.34E+06	2.10E+06		385847
Diff in mean cf Adult			-288700	-17280	215000	759200	1372000	1115000		
SE of diff			45400	114400	144600	243800	319000	296200		
95% CI diff		from	-385000	-250100	-80220	253500	687600	414200		
		to	-192500	215500	510200	1265000	2056000	1815000		
P			<0.0001	0.88	0.15	0.0051	0.0007	0.0070		
**NK Cells Fig 2E**										
Number of values	22		30	26	26	22	15	8		17
Mean	690758		198246	274998	337896	402654	409671	371387		242938
Std. Deviation	654202		178439	233166	267169	379246	384614	135029		132478
Std. Error	139476		32578	45728	52396	80856	99307	47740		32131
Lower 95% CI of mean	400701		131615	180820	229985	234505	196677	258498		174824
Upper 95% CI of mean	980816		264877	369176	445808	570803	622665	484275		311052
Diff in mean cf Adult			-44690	32060	94960	159700	166700	128400		
SE of diff			45760	55890	61460	87010	104400	57550		
95% CI diff		from	-137100	-80890	-29520	-18820	-54540	4150		
		to	47740	145000	219400	338300	388000	252700		
P			0.33	0.57	0.13	0.077	0.13	0.044		
**NKT Cells Fig 2F**										
Number of values	20		30	26	26	22	15	8		17
Mean	55452		1803	4691	12601	49832	26002	17788		33691
Std. Deviation	55471		4714	8507	21142	163664	51892	15590		17249
Std. Error	12404		860.7	1668	4146	34893	13399	5512		4183
Lower 95% CI of mean	29491		42.45	1255	4062	-22733	-2736	4754		24822
Upper 95% CI of mean	81414		3563	8127	21141	122397	54739	30821		42559
Diff in mean cf Adult			-32000	-29000	-21000	16000	-7700	-16000		
SE of diff			4300	4500	5900	35000	14000	6900		
95% CI diff		from	-41000	-38000	-33000	-57000	-37000	-31000		
		to	-23000	-20000	-9200	89000	22000	-1200		
P			<0.0001	<0.0001	0.001	0.65	0.59	0.036		
**MDC Fig 2G**										
Number of values	6		21	18	21	19	15	6		10
Mean	10746		7162	14977	14083	16910	16593	23894		9435
Std. Deviation	8650		7719	18456	13380	13603	12607	9319		7485
Std. Error	3531		1684	4350	2920	3121	3255	3804		2367
Lower 95% CI of mean	1669		3648	5799	7993	10354	9611	14115		4080
Upper 95% CI of mean	19824		10676	24155	20174	23467	23574	33674		14790
Diff in mean cf Adult			-2273	5542	4648	7475	7158	14460		
SE of diff			2905	4952	3759	3917	4025	4481		
95% CI diff		from	-8377	-4680	-3050	-577.7	-1190	4127		
		to	3831	15760	12350	15530	15510	24790		
P			0.44	0.27	0.23	0.067	0.089	0.012		
**PDC Fig 2H**										
Number of values	7		21	18	21	19	15	6		10
Mean	14978		3538	11049	7890	12657	12273	16797		13947
Std. Deviation	14560		4539	26350	9722	11756	11343	11098		10315
Std. Error	5503		990.4	6211	2122	2697	2929	4531		3262
Lower 95% CI of mean	1512		1471	-2054	3465	6991	5991	5150		6568
Upper 95% CI of mean	28444		5604	24153	12316	18324	18555	28443		21326
Diff in mean cf Adult			-10410	-2898	-6056	-1289	-1674	2850		
SE of diff			3409	7015	3891	4232	4384	5583		
95% CI diff			-18000	-17380	-14310	-10120	-10820	-9588		
			-2814	11580	2192	7539	7471	15290		
P			0.012	0.68	0.14	0.76	0.71	0.62		
