# Supplementary material for: Immune reconstitution following umbilical cord blood transplantation: IRES, a study of UK paediatric patients
Source: EJHaem. 2020 May 21;1(1):208–18. doi: 10.1002/jha2.12 (PMC9176140; doi:10.1002/jha2.12)
Supplement: Supplementary file 2 — SUPPORTING INFORMATION [file JHA2-1-208-s006.pdf]

	Sample	1		2		3		6		12		Month
**T Cells**	**Fig 3A**	**-**	**+**	**-**	**+**	**-**	**+**	**-**	**+**	**-**	**+**	**ATG**
Number of values		15	13	11	12	14	9	11	7	8	6	
Mean		229450	72850	430357	208767	764430	113118	2.60E+06	367855	2.96E+06	2.30E+06	
Std. Deviation		189516	152533	635812	585618	789611	122827	2.95E+06	371862	2.17E+06	1.45E+06	
Std. Error		48933	42305	191705	169053	211032	40942	889123	140550	767542	589941	
Lower 95% CI of mean		124498	-19324	3216	-163313	308522	18705	623287	23938	1.14E+06	787666	
Upper 95% CI of mean		334402	165025	857497	580846	1.22E+06	207530	4.59E+06	711772	4.77E+06	3.82E+06	
Diff in mean	cf +ATG	156600		221600		651300		2237000		651900		
SE of diff		64690		255600		215000		900200		968100		
95% CI diff	from	23350		-311600		187000		230900		-1479000		
	to	289900		754800		1116000		4242000		2783000		
P		0.023		0.4		0.0097		0.032		0.52		

**B Cells**	**Fig 3B**	-	+	-	+	-	+	-	+	-	+	ATG
Number of values		15	13	11	12	14	9	11	7	8	6	
Mean		1071	855.2	366253	233197	666202	137231	1.27E+06	723005	1.79E+06	1.31E+06	
Std. Deviation		2622	3083	742024	355698	667082	158610	1.37E+06	911554	1.37E+06	1.07E+06	
Std. Error		677.1	855.2	223729	102681	178285	52870	413276	344535	485670	437385	
Lower 95% CI of mean		-381.4	-1008	-132241	7200	281040	15313	346524	-120048	643692	186687	
Upper 95% CI of mean		2523	2718	864747	459194	1.05E+06	259149	2.19E+06	1.57E+06	2.94E+06	2.44E+06	
Diff in mean	cf +ATG	220		130000		530000		540000		480000		
se of diff		1100		250000		190000		540000		650000		
95% CI diff	from	-2000		-390000		130000		-600000		-960000		
	to	2500		660000		930000		1700000		1900000		
P		0.85		0.6		0.012		0.33		0.48		
