# Supplementary material for: Immune reconstitution following umbilical cord blood transplantation: IRES, a study of UK paediatric patients
Source: EJHaem. 2020 May 21;1(1):208–18. doi: 10.1002/jha2.12 (PMC9176140; doi:10.1002/jha2.12)
Supplement: Supplementary file 3 — SUPPORTING INFORMATION [file JHA2-1-208-s009.pdf]

| Sample | Cord | 1 | 2 | 3 | 6 | 12 | 18-24 | Month | Adult |
|---|---|---|---|---|---|---|---|---|---|
| CD3+CD4+CD11+ Fig 5C | | | | | | | | | |
| Number of values | 20 | 17 | 15 | 21 | 18 | 13 | 7 | | 19 |
| Mean | 0.4935 | 5.952 | 15.43 | 9.511 | 5.745 | 2.816 | 5.203 | | 3.047 |
| Std. Deviation | 0.3304 | 6.597 | 21.1 | 12.11 | 8.182 | 3.756 | 5.324 | | 2.408 |
| Std. Error | 0.07388 | 1.6 | 5.449 | 2.643 | 1.928 | 1.042 | 2.012 | | 0.5524 |
| Lower 95% CI of mean | 0.3389 | 2.561 | 3.744 | 3.998 | 1.676 | 0.5464 | 0.2785 | | 1.887 |
| Upper 95% CI of mean | 0.6481 | 9.344 | 27.12 | 15.02 | 9.814 | 5.086 | 10.13 | | 4.208 |
| Diff in mean cf Adult | | 2.905 | 12.38 | 6.464 | 2.698 | -0.2312 | 2.155 | | |
| SE of diff | | 1.693 | 5.477 | 2.7 | 2.006 | 1.179 | 2.087 | | |
| 95% CI diff from | | -0.6376 | 0.6353 | 0.8474 | -1.501 | -2.709 | -2.951 | | |
| to | | 6.448 | 24.13 | 12.08 | 6.896 | 2.246 | 7.262 | | |
| P | | 0.1 | 0.04 | 0.026 | 0.2 | 0.85 | 0.34 | | |
| | | | | | | | | | |
| CD3+CD4-CD161+ Fig 5D | | | | | | | | | |
| Number of values | 20 | 19 | 16 | 21 | 17 | 13 | 7 | | 19 |
| Mean | 4.839 | 3.047 | 5.698 | 3.982 | 3.841 | 3.915 | 3.777 | | 10.75 |
| Std. Deviation | 2.366 | 2.408 | 3.73 | 6.227 | 3.527 | 3.797 | 2.216 | | 7.714 |
| Std. Error | 0.5292 | 0.5524 | 0.9325 | 1.359 | 0.8554 | 1.053 | 0.8375 | | 1.77 |
| Lower 95% CI of mean | 3.731 | 1.887 | 3.71 | 1.148 | 2.027 | 1.62 | 1.728 | | 7.034 |
| Upper 95% CI of mean | 5.946 | 4.208 | 7.685 | 6.817 | 5.654 | 6.209 | 5.826 | | 14.47 |
| Diff in mean cf Adult | | -7.705 | -5.055 | -6.77 | -6.912 | -6.838 | -6.975 | | |
| SE of diff | | 1.854 | 2 | 2.231 | 1.966 | 2.059 | 1.958 | | |
| 95% CI diff from | | -11.56 | -9.168 | -11.31 | -10.96 | -11.06 | -11.03 | | |
| to | | -3.849 | -0.9422 | -2.233 | -2.863 | -2.612 | -2.924 | | |
| P | | 0.0004 | 0.018 | 0.0046 | 0.0017 | 0.0026 | 0.0017 | | |
| | | | | | | | | | |
| CD3+CD57+ Fig 5E | | | | | | | | | |
| Number of values | 21 | 16 | 15 | 21 | 19 | 15 | 7 | | 19 |
| Mean | 0.1629 | 5.548 | 4.513 | 6.565 | 5.372 | 8.039 | 0.9157 | | 5.864 |
| Std. Deviation | 0.1102 | 10.44 | 4.314 | 8.533 | 10.26 | 18.16 | 0.9373 | | 4.978 |
| Std. Error | 0.02405 | 2.61 | 1.114 | 1.862 | 2.354 | 4.69 | 0.3543 | | 1.142 |
| Lower 95% CI of mean | 0.1127 | -0.01388 | 2.124 | 2.68 | 0.4262 | -2.02 | 0.04887 | | 3.464 |
| Upper 95% CI of mean | 0.213 | 11.11 | 6.902 | 10.45 | 10.32 | 18.1 | 1.783 | | 8.263 |
| Diff in mean cf Adult | | -0.3156 | -1.351 | 0.7011 | -0.4916 | 2.175 | -4.948 | | |
| SE of diff | | 2.848 | 1.595 | 2.184 | 2.617 | 4.827 | 1.196 | | |
| 95% CI diff from | | -6.257 | -4.605 | -3.75 | -5.871 | -8.111 | -7.435 | | |
| to | | 5.626 | 1.903 | 5.153 | 4.888 | 12.46 | -2.461 | | |
| P | | 0.91 | 0.4 | 0.75 | 0.85 | 0.66 | 0.0005 | | |
| | | | | | | | | | |
| Treg Fig 2F | | | | | | | | | |
| Number of values | 22 | 19 | 17 | 22 | 20 | 14 | 8 | | 20 |
| Mean | 6.305 | 8.773 | 10.02 | 7.301 | 9.081 | 6.915 | 8.858 | | 4.951 |
| Std. Deviation | 1.524 | 5.712 | 6.224 | 3.826 | 4.864 | 3.008 | 4.668 | | 2.261 |
| Std. Error | 0.3248 | 1.31 | 1.51 | 0.8158 | 1.088 | 0.8039 | 1.65 | | 0.5055 |
| Lower 95% CI of mean | 5.629 | 6.019 | 6.817 | 5.605 | 6.805 | 5.178 | 4.956 | | 3.892 |
| Upper 95% CI of mean | 6.98 | 11.53 | 13.22 | 8.998 | 11.36 | 8.652 | 12.76 | | 6.009 |
| Diff in mean cf Adult | | 3.822 | 5.067 | 2.351 | 4.131 | 1.964 | 3.908 | | |
| SE of diff | | 1.405 | 1.592 | 0.9597 | 1.199 | 0.9497 | 1.726 | | |
| 95% CI diff from | | 0.916 | 1.735 | 0.399 | 1.665 | -0.00532 | -0.07238 | | |
| to | | 6.728 | 8.399 | 4.302 | 6.597 | 3.934 | 7.888 | | |
| P | | 0.012 | 0.0049 | 0.02 | 0.002 | 0.051 | 0.053 | | |
