# Supplementary material for: Immune reconstitution following umbilical cord blood transplantation: IRES, a study of UK paediatric patients
Source: EJHaem. 2020 May 21;1(1):208–18. doi: 10.1002/jha2.12 (PMC9176140; doi:10.1002/jha2.12)
Supplement: Supplementary file 4 — SUPPORTING INFORMATION [file JHA2-1-208-s001.pdf]

Sample	Cord	1	2	3	6	12	18-24	Month	Adult
TrB Fig 6A									
Number of values	19.0		11.0	18.0	20.0	15.0	8.0		19.0
Mean	21.1		38.9	45.9	25.4	14.8	14.2		4.5
Std. Deviation	4.8		34.8	33.6	26.5	9.8	5.0		2.2
Std. Error	1.1		10.5	7.9	5.9	2.5	1.8		0.5
Lower 95% CI of mean	18.8		15.5	29.2	13.0	9.4	10.0		3.4
Upper 95% CI of mean	23.4		62.3	62.7	37.8	20.2	18.4		5.6
Diff in mean cf Adult		NA	34.4	41.4	20.9	10.3	9.7		
SE of diff			10.5	7.9	5.9	2.6	1.9		
95% CI diff from			11.0	24.7	8.4	4.8	5.4		
to			57.8	58.2	33.3	15.8	14.0		
P			0.0084	<0.0001	0.0023	0.0012	0.0008		
CD19+CD27+ Fig 6B									
Number of values	22.0		10.0	17.0	16.0	15.0	8.0		17.0
Mean	1.1		1.1	3.8	4.8	7.8	6.8		17.0
Std. Deviation	0.8		1.5	5.3	2.7	6.4	2.2		5.6
Std. Error	0.2		0.5	1.3	0.7	1.7	0.8		1.4
Lower 95% CI of mean	0.7		0.1	1.0	3.3	4.3	4.9		14.2
Upper 95% CI of mean	1.5		2.2	6.5	6.3	11.4	8.7		19.9
Diff in mean cf Adult		NA	-15.9	-13.3	-12.2	-9.2	-10.3		
SE of diff			1.4	1.9	1.5	2.1	1.6		
95% CI diff from			-18.9	-17.1	-15.4	-13.6	-13.5		
to			-12.9	-9.5	-9.1	-4.8	-7.0		
P			<0.0001	<0.0001	<0.0001	0.0002	<0.0001		
