# Supplementary material for: Immune reconstitution following umbilical cord blood transplantation: IRES, a study of UK paediatric patients
Source: EJHaem. 2020 May 21;1(1):208–18. doi: 10.1002/jha2.12 (PMC9176140; doi:10.1002/jha2.12)
Supplement: Supplementary file 5 — SUPPORTING INFORMATION [file JHA2-1-208-s002.pdf]

Sample	Cord	1	2	3	6	12	18-24	Month	Adult
CD14+ 16+ Fig 7A									
Number of values	24.0	29.0	26.0	26.0	22.0	15.0	8.0		17.0
Mean	13.4	29.9	23.3	21.0	16.2	14.7	13.0		14.5
Std. Deviation	9.0	15.8	12.6	12.9	8.5	9.3	5.7		7.3
Std. Error	1.8	2.9	2.5	2.5	1.8	2.4	2.0		1.8
Lower 95% CI of mean	9.6	23.9	18.2	15.8	12.4	9.6	8.2		10.8
Upper 95% CI of mean	17.2	35.9	28.4	26.2	19.9	19.9	17.8		18.3
Diff in mean cf Adult		15.3	8.7	6.5	1.6	0.2	-1.5		
SE of diff		3.4	3.0	3.1	2.5	3.0	2.7		
95% CI diff from		8.4	2.6	0.2	-3.5	-5.9	-7.2		
to		22.2	14.9	12.7	6.8	6.3	4.1		
P		<0.0001	0.0065	0.0425	0.52	0.95	0.58		
CD56++ Fig 7B									
Number of values	22.0	28.0	26.0	26.0	22.0	15.0	8.0		17.0
Mean	16.2	27.8	41.5	34.6	25.5	14.0	18.5		6.5
Std. Deviation	22.4	24.8	27.4	24.0	20.1	13.4	22.7		4.4
Std. Error	4.8	4.7	5.4	4.7	4.3	3.5	8.0		1.1
Lower 95% CI of mean	6.3	18.1	30.5	24.9	16.6	6.6	-0.5		4.3
Upper 95% CI of mean	26.2	37.4	52.6	44.3	34.4	21.4	37.5		8.8
Diff in mean cf Adult		21.2	35.0	28.1	19.0	7.5	12.0		
SE of diff		4.8	5.5	4.8	4.4	3.6	8.1		
95% CI diff from		11.4	23.7	18.2	9.8	-0.2	-7.2		
to		31.0	46.3	38.0	28.1	15.1	31.1		
P		0.0001	<0.0001	<0.0001	0.0003	0.056	0.18		
CD56+CD16+CD62L+ Fig7C									
Number of values	21.0	26.0	24.0	24.0	21.0	14.0	7.0		17.0
Mean	3.8	38.0	27.9	23.1	18.0	20.3	18.6		3.9
Std. Deviation	3.2	23.0	21.4	18.7	14.2	15.8	8.3		2.3
Std. Error	0.7	4.5	4.4	3.8	3.1	4.2	3.1		0.6
Lower 95% CI of mean	2.4	28.8	18.9	15.3	11.5	11.2	10.9		2.7
Upper 95% CI of mean	5.3	47.3	37.0	31.0	24.4	29.4	26.3		5.1
Diff in mean cf Adult		34.2	24.1	19.3	14.1	16.4	14.8		
SE of diff		4.5	4.4	3.9	3.2	4.3	3.2		
95% CI diff from		24.8	15.0	11.3	7.5	7.2	6.9		
to		43.5	33.2	27.2	20.7	25.6	22.6		
P		<0.0001	<0.0001	<0.0001	0.0002	0.002	0.0036		
CD56+CD16+CD57+ Fig 7D									
Number of values	21.0	26.0	24.0	23.0	20.0	14.0	7.0		19.0
Mean	1.2	16.5	11.6	12.1	10.1	17.3	4.3		23.0
Std. Deviation	0.6	18.7	10.8	10.6	12.6	20.8	3.4		12.3
Std. Error	0.1	3.7	2.2	2.2	2.8	5.6	1.3		2.8
Lower 95% CI of mean	0.9	9.0	7.0	7.5	4.2	5.3	1.1		17.1
Upper 95% CI of mean	1.4	24.1	16.2	16.7	16.0	29.2	7.5		29.0
Diff in mean cf Adult		-6.5	-11.4	-10.9	-12.9	-5.8	-18.7		
SE of diff		4.6	3.6	3.6	4.0	6.2	3.1		
95% CI diff from		-15.9	-18.7	-18.2	-21.0	-18.8	-25.1		
to		2.8	-4.1	-3.6	-4.8	7.3	-12.3		
P		0.17	0.003	0.0044	0.0026	0.37	<0.0001		
