# Supplementary material for: Immune reconstitution following umbilical cord blood transplantation: IRES, a study of UK paediatric patients
Source: EJHaem. 2020 May 21;1(1):208–18. doi: 10.1002/jha2.12 (PMC9176140; doi:10.1002/jha2.12)
Supplement: Supplementary file 6 — SUPPORTING INFORMATION [file JHA2-1-208-s003.pdf]

	1		2		3		6		12		Month
CD3+CD4-CD45RA+CD31+ Fig 8A	-	+	-	+	-	+	-	+	-	+	Median Age
Number of values	9	13	7	10	10	13	10	10	11	4	
Mean	0.53	0.27	0.55	0.20	0.60	0.26	0.69	0.35	0.62	0.66	
Std. Deviation	0.27	0.24	0.23	0.17	0.23	0.19	0.23	0.30	0.16	0.25	
Std. Error	0.09	0.07	0.09	0.05	0.07	0.05	0.07	0.09	0.05	0.13	
Lower 95% CI of mean	0.32	0.12	0.34	0.08	0.44	0.15	0.52	0.14	0.51	0.26	
Upper 95% CI of mean	0.74	0.41	0.76	0.33	0.77	0.38	0.86	0.57	0.73	1.07	
Diff in mean Cf >median	0.27		0.34		0.34		0.33		-0.04		
SE of diff	0.11		0.10		0.09		0.12		0.14		
95% CI diff from	0.03		0.12		0.15		0.08		-0.47		
to	0.51		0.57		0.53		0.59		0.39		
P	0.032		0.0069		0.0018		0.014		0.79		
CD4:CD8 Fig 8B											
Number of values	9	11	7	10	10	13	10	10	11	4	
Mean	4.51	4.44	5.11	3.31	4.83	2.87	5.91	1.65	2.53	1.40	
Std. Deviation	3.71	2.97	4.43	3.01	2.64	3.88	3.31	0.87	1.31	0.78	
Std. Error	1.24	0.90	1.68	0.95	0.84	1.08	1.05	0.28	0.40	0.39	
Lower 95% CI of mean	1.66	2.45	1.01	1.15	2.94	0.52	3.54	1.03	1.64	0.16	
Upper 95% CI of mean	7.36	6.44	9.21	5.46	6.72	5.21	8.27	2.27	3.41	2.65	
Diff in mean Cf >median	0.07		1.80		1.97		4.26		1.12		
SE of diff	1.53		1.93		1.36		1.08		0.56		
95% CI diff from	3.32		-2.56		-0.87		1.85		-0.14		
to			6.16		4.81		6.67		2.38		
P	0.97		0.37		0.16		0.0028		0.074		
TrB Fig 8C											
Number of values	too few		3	8	7	11	8	11	11	4	
Mean			10.54	49.58	54.38	40.58	31.37	23.32	18.18	5.53	
Std. Deviation			0.80	35.44	27.72	37.13	28.04	26.24	9.14	3.50	
Std. Error			0.46	12.53	10.48	11.19	9.91	7.91	2.75	1.75	
Lower 95% CI of mean			8.54	19.95	28.75	15.63	7.93	5.70	12.04	-0.04	
Upper 95% CI of mean			12.53	79.22	80.02	65.52	54.81	40.95	24.31	11.11	
Diff in mean Cf >median			-39.05		13.81		8.05		12.64		
SE of diff			12.54		15.33		12.68		3.26		
95% CI diff from			-68.70		-18.87		-19.15		5.53		
to			-9.39		46.48		35.25		19.76		
P			0.017		0.38		0.54		0.0022		
