# Supplementary material for: Immune reconstitution following umbilical cord blood transplantation: IRES, a study of UK paediatric patients
Source: EJHaem. 2020 May 21;1(1):208–18. doi: 10.1002/jha2.12 (PMC9176140; doi:10.1002/jha2.12)
Supplement: Supplementary file 7 — SUPPORTING INFORMATION [file JHA2-1-208-s004.pdf]

	Sample	1		2		3		6		12		Month
CD3	Supp Fig 1A	-	+	-	+	-	+	-	+	-	+	Median Age
Number of values		10	20	10	16	11	15	10	12	11	4	
Mean		298663	82479	365736	301848	330361	719343	1.97E+06	1.33E+06	3.29E+06	1.18E+06	
Std. Deviation		209553	114271	698920	500051	286415	865061	2.25E+06	2.42E+06	1.73E+06	887228	
Std. Error		66266	25552	221018	125013	86357	223358	710025	698738	521735	443614	
Lower 95% CI of mean		148757	28998	-134245	35391	137947	240283	360892	-209376	2.13E+06	-235287	
Upper 95% CI of mean		448570	135961	865718	568305	522776	1.20E+06	3.57E+06	2.87E+06	4.45E+06	2.59E+06	
Diff in mean	Cf >median	216200		63890		-389000		638600		2113000		
SE of diff		71020		253900		239500		996200		684800		
95% CI diff	from	59860		-480800		-894300		-1446000		587500		
	to	372500		608600		116300		2724000		3639000		
P		0.0112		0.81		0.12		0.53		0.012		
Granulocyte	Supp Fig1B											
Number of values		10	20	10	16	11	15	10	12	11	4	
Mean		2.85E+06	1.57E+06	5.21E+06	5.13E+06	5.03E+06	7.38E+06	5.30E+06	2.92E+06	5.05E+06	1.78E+06	
Std. Deviation		2.28E+06	1.47E+06	3.20E+06	6.36E+06	4.52E+06	1.16E+07	2.19E+06	1.56E+06	3.61E+06	975957	
Std. Error		719404	328503	1.01E+06	1.59E+06	1.36E+06	2.99E+06	692069	450819	1.09E+06	487978	
Lower 95% CI of mean		1.22E+06	877342	2.92E+06	1.74E+06	2.00E+06	971741	3.74E+06	1.93E+06	2.63E+06	231250	
Upper 95% CI of mean		4.47E+06	2.25E+06	7.50E+06	8.52E+06	8.06E+06	1.38E+07	6.87E+06	3.92E+06	7.48E+06	3.34E+06	
Diff in mean	Cf >median	1281000		81380		-2346000		2378000		3269000		
SE of diff		790900		1884000		3282000		826000		1193000		
95% CI diff	from	-442300		-3817000		-9215000		618100		668600		
	to	3004000		3980000		4523000		4138000		5870000		
P		0.13		0.97		0.48		0.012		0.018		
NK Cells	Supp Fig1C											
Number of values		10	20	10	16	11	15	10	12	11	4	
Mean		251587	93250	249845	119672	252252	222024	415175	215685	470652	115673	
Std. Deviation		217891	87562	268134	91390	204680	222539	432417	132114	389468	64094	
Std. Error		68903	19579	84791	22848	61713	57459	136742	38138	117429	32047	
Lower 95% CI of mean		95716	52269	58032	70974	114747	98785	105841	131745	209007	13684	
Upper 95% CI of mean		407459	134230	441658	168370	389756	345263	724510	299624	732298	217661	
Diff in mean	Cf >median	158300		130200		30230		199500		355000		
SE of diff		71630		87820		84320		142000		121700		
95% CI diff	from	-1256		-65480		-144700		-116800		87070		
	to	317900		325800		205100		515800		622900		
P		0.052		0.17		0.72		0.19		0.014		
B Cells	Supp Fig 1D											
Number of values		10	20	10	16	11	15	10	12	11	4	
Mean		1497	610.4	360321	217343	475768	525763	1.43E+06	728482	2.00E+06	721650	
Std. Deviation		3167	2481	795058	299663	739048	695312	1.47E+06	633084	1.23E+06	592321	
Std. Error		1002	554.7	251419	74916	222831	179529	464277	182756	370161	296160	
Lower 95% CI of mean		-768.5	-550.7	-208434	57664	-20727	140709	383018	326245	1.18E+06	-220870	
Upper 95% CI of mean		3763	1771	929076	377021	972263	910818	2.48E+06	1.13E+06	2.83E+06	1.66E+06	
Diff in mean	Cf >median	886.8		143000		-50000		704800		1282000		
SE of diff		1145		262300		286200		499000		474100		
95% CI diff	from	-1569		-441500		-646900		-393400		238300		
	to	3343		727500		546900		1803000		2325000		
P		0.45		0.6		0.86		0.19		0.021		
