# Supplementary material for: Immune reconstitution following umbilical cord blood transplantation: IRES, a study of UK paediatric patients
Source: EJHaem. 2020 May 21;1(1):208–18. doi: 10.1002/jha2.12 (PMC9176140; doi:10.1002/jha2.12)
Supplement: Supplementary file 8 — SUPPORTING INFORMATION [file JHA2-1-208-s010.pdf]

		Cord	1	2	3	6	12	18-24	Month	Adult
CD4:CD8	Supp Fig 2B									
Number of values		22	20	17	23	20	15	8		19
Mean		2.8	4.5	4.0	3.7	3.8	2.2	2.3		2.9
Std. Deviation		0.8	3.2	3.6	3.5	3.2	1.3	1.0		1.4
Std. Error		0.2	0.7	0.9	0.7	0.7	0.3	0.3		0.3
Lower 95% CI of mean		2.5	3.0	2.2	2.2	2.3	1.5	1.4		2.2
Upper 95% CI of mean		3.2	6.0	5.9	5.2	5.3	2.9	3.1		3.6
Diff in mean	cf Adult		1.6	1.1	0.8	0.9	-0.7	-0.7		
SE of diff			0.8	0.9	0.8	0.8	0.5	0.5		
95% CI diff	from		-0.1	-0.8	-0.8	-0.8	-1.6	-1.7		
	to		3.2	3.1	2.4	2.5	0.3	0.3		
P			0.061	0.25	0.32	0.29	0.15	0.18		

		1		2		3		6		12		Month
CD4:CD8	Supp Fig 2C	-	+	-	+	-	+	-	+	-	+	ATG
Number of values		13	4	11	3	14	6	11	5	8	6	
Mean		5.535	1.975	5.212	1.497	3.864	4.393	3.876	4.958	2.399	2.048	
Std. Deviation		3.337	2.063	3.969	1.656	3.927	2.739	3.014	4.057	1.525	1.088	
Std. Error		0.9254	1.032	1.197	0.9558	1.05	1.118	0.9089	1.815	0.5392	0.444	
Lower 95% CI of mean		3.518	-1.308	2.546	-2.616	1.597	1.519	1.851	-0.07996	1.124	0.9069	
Upper 95% CI of mean		7.551	5.258	7.878	5.609	6.132	7.268	5.901	9.996	3.674	3.19	
Diff in mean	cf +ATG	3.56		3.715		-0.529		-1.082		0.3504		
SE of diff		1.386		1.531		1.534		2.029		0.6985		
95% CI diff	from	0.3637		0.1835		-3.842		-6.048		-1.187		
	to	6.756		7.247		2.784		3.884		1.888		
P		0.033		0.042		0.74		0.61		0.63		
