# Supplementary material for: Immune reconstitution following umbilical cord blood transplantation: IRES, a study of UK paediatric patients
Source: EJHaem. 2020 May 21;1(1):208–18. doi: 10.1002/jha2.12 (PMC9176140; doi:10.1002/jha2.12)
Supplement: Supplementary file 9 — SUPPORTING INFORMATION [file JHA2-1-208-s005.pdf]

		1		2		3		6		Month
CD56+CD16+	Supp Fig 3A	-	+	-	+	-	+	-	+	Survival
Number of values		8	22	4	22	5	21	4	18	
Mean		190417	129888	56842	190265	69149	274256	94863	353362	
Std. Deviation		169603	156943	80886	194782	64786	215799	19346	331850	
Std. Error		59964	33460	40443	41528	28973	47091	9673	78218	
Lower 95% CI of mean		48623	60303	-71866	103904	-11293	176025	64079	188336	
Upper 95% CI of mean		332210	199473	185551	276627	149592	372487	125647	518388	
Diff in mean	cf Survival	60530		-133400		-205100		-258500		
se of diff		68670		57970		55290		78810		
95% CI diff	from	-90610		-262600		-319800		-424800		
	to	211700		-4272		-90430		-92200		
P		0.4		0.044		0.0012		0.0044		
PDC	Supp Fig 3B									
Number of values		8	21	3	18	4	19	3	16	
Mean		3406	2240	1131	10861	1918	8317	1607	14729	
Std. Deviation		3588	4396	1720	26422	1379	10174	700.4	11695	
Std. Error		1269	959.3	993.2	6228	689.7	2334	404.4	2924	
Lower 95% CI of mean		405.9	239	-3142	-2279	-277.3	3414	-133.2	8497	
Upper 95% CI of mean		6406	4241	5405	24000	4112	13221	3347	20961	
Diff in mean		1166		-9729		-6400		-13120		
se of diff		1591		6306		2434		2952		
95% CI diff		-2224		-23040		-11480		-19410		
		4555		3577		-1323		-6833		
P		0.48		0.14		0.016		0.0005		
MDC	Supp Fig 3C									
Number of values		8	20	3	18	4	19	3	16	
Mean		6057	4422	6317	13924	8522	13772	7925	18595	
Std. Deviation		5278	7970	5472	19034	9704	14082	6772	14027	
Std. Error		1866	1782	3159	4486	4852	3231	3910	3507	
Lower 95% CI of mean		1645	691.7	-7277	4458	-6919	6984	-8897	11120	
Upper 95% CI of mean		10469	8152	19911	23389	23963	20559	24747	26069	
Diff in mean		1635		-7606		-5250		-10670		
se of diff		2580		5487		5829		5252		
95% CI diff		-3765		-19560		-19510		-24170		
		7036		4350		9014		2833		
P		0.53		0.19		0.4		0.1		
Monocytes	Supp Fig 3D									
Number of values		8	22	4	22	5	21	4	18	
Mean		665447	640941	1.42E+06	1.12E+06	923621	1.52E+06	960531	1.01E+06	
Std. Deviation		439992	656047	1.07E+06	834125	742088	1.54E+06	191340	668986	
Std. Error		155561	139870	534285	177836	331872	336787	95670	157682	
Lower 95% CI of mean		297600	350065	-280892	752907	2192	812138	656065	672746	
Upper 95% CI of mean		1.03E+06	931816	3.12E+06	1.49E+06	1.85E+06	2.22E+06	1.27E+06	1.34E+06	
Diff in mean		24510		296700		-591000		-44900		
se of diff		209200		563100		472800		184400		
95% CI diff		-415000		-1495000		-1612000		-434100		
		464000		2089000		430300		344300		
P		0.91		0.64		0.23		0.81		
