# Supplementary material for: Immune reconstitution following umbilical cord blood transplantation: IRES, a study of UK paediatric patients
Source: EJHaem. 2020 May 21;1(1):208–18. doi: 10.1002/jha2.12 (PMC9176140; doi:10.1002/jha2.12)
Supplement: Supplementary file 10 — SUPPORTING INFORMATION [file JHA2-1-208-s008.pdf]

Supplementary Figures

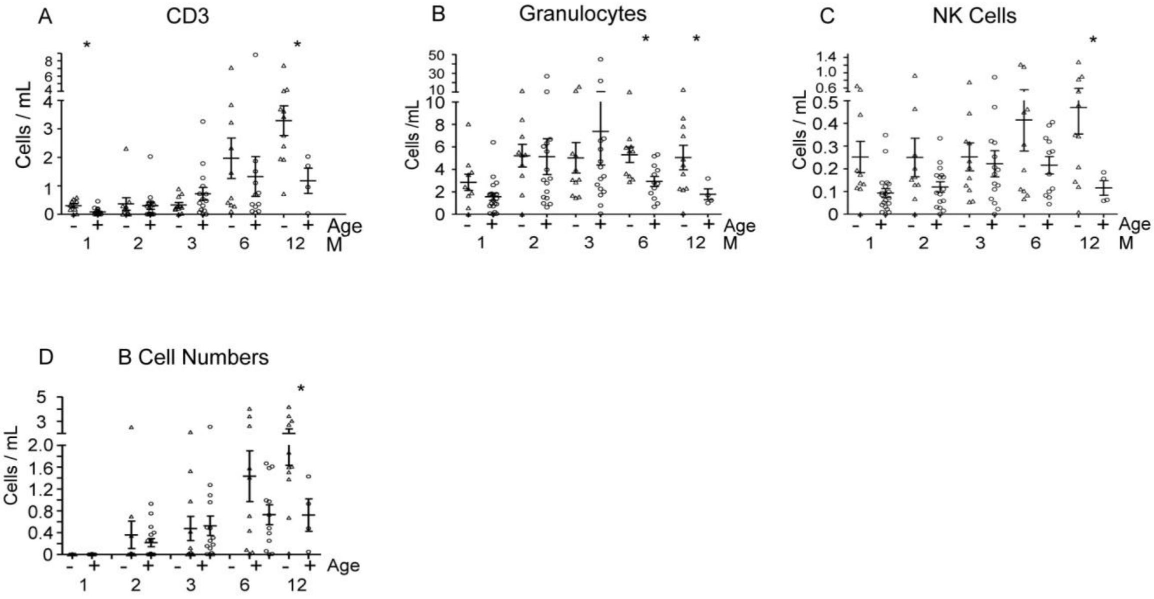

Supp fig 1. Absolute cell levels according to median age. (a) CD3, (b) granulocytes and (c) CD56+CD16+ NK cells (in 10^6 cells/mL) by median age (- below, + above) at 1 to 12 months post-transplant. Asterisks: P<0.05. Further statistical information in Supp Table 7.

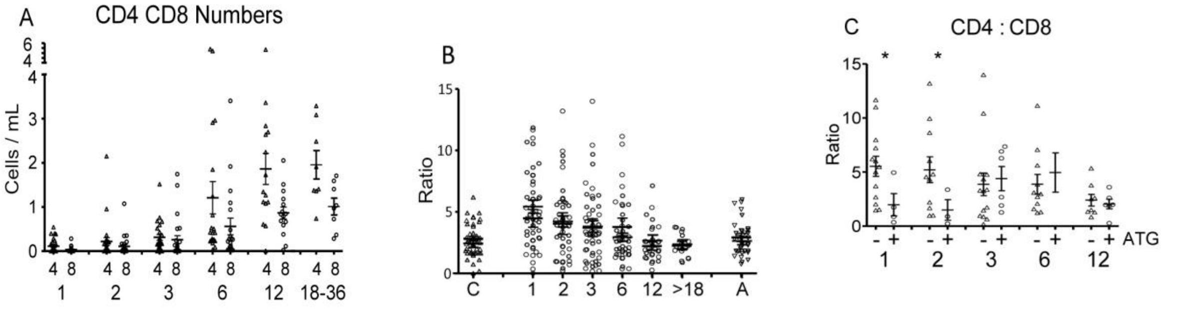

Supp fig 2. CD4 and CD8 T subsets. (a) Absolute cell levels (in 10^6 cells/mL) of CD4 (4) and CD8 (8) T cells at months post-transplant for all patients (, (b) CD4:CD8 ratios for all patients, (c) CD4:CD8 ratios according to ATG treatment. Bars represent means with standard errors, asterisks: P<0.05. Further statistical information in Supp Table 8.

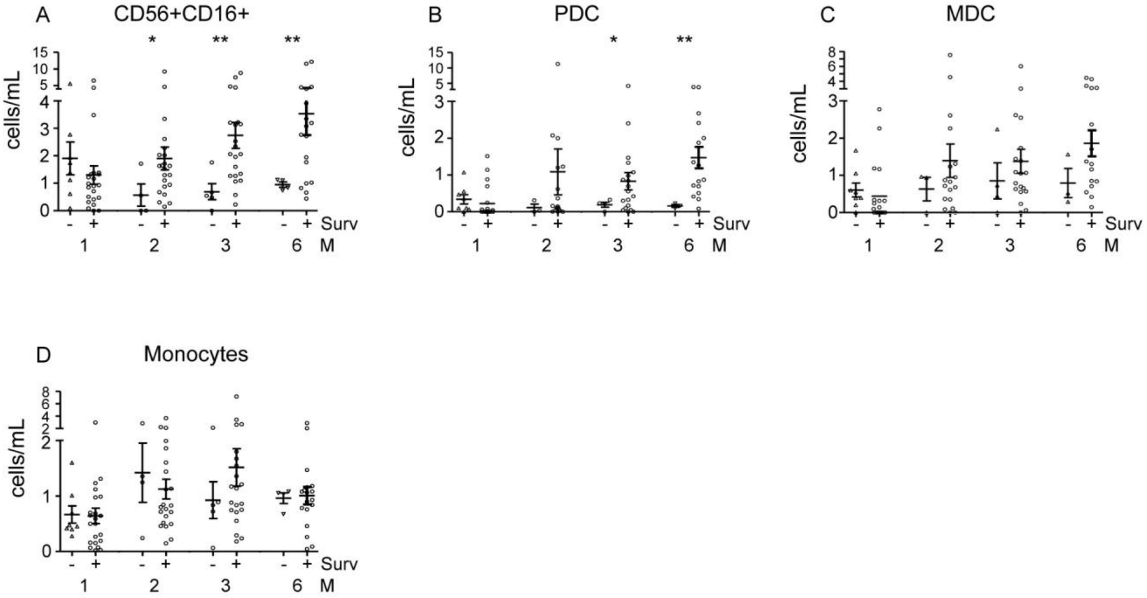

Supp fig 3. One year survival correlations. Numbers of (A) CD56+16+ NK cells ($x10^5$), (B) PDC ($x10^4$), (C) MDC ($x10^4$) and (D) CD14+ monocytes ($x10^6$) are shown according to survival (Surv +) or not (-) at months (M) after transplant. For '-', 1M n=8, 2M n=4, 3M n=5, 6M n=4. (* P<0.05, ** P<0.005.) Further statistical information in Supp Table 9.
